# Effects of down-regulating ornithine decarboxylase upon putrescine-associated metabolism and growth in *Nicotiana tabacum* L.

**DOI:** 10.1093/jxb/erw166

**Published:** 2016-04-28

**Authors:** Heidi L. Dalton, Cecilia K. Blomstedt, Alan D. Neale, Ros Gleadow, Kathleen D. DeBoer, John D. Hamill

**Affiliations:** ^1^School of Biological Sciences, Monash University, Melbourne, Victoria 3800, Australia; ^2^Department of Molecular Ecology, Max Planck Institute for Chemical Ecology, 07745 Jena, Germany; ^3^Deakin University, Centre for Regional and Rural Futures (CeRRF), Geelong, Victoria 3216, Australia; ^4^The UWA Institute of Agriculture, The University of Western Australia, Crawley, WA 6009, Australia

**Keywords:** Alkaloid, gene expression, ODC, phenolamide, polyamine, putrescine, PMT, QPT, RNAi.

## Abstract

Transgenic plants of *Nicotiana tabacum* L. homozygous for an RNAi construct designed to silence *ornithine decarboxylase* (*ODC*) had significantly lower concentrations of nicotine and nornicotine, but significantly higher concentrations of anatabine, compared with vector-only controls. Silencing of *ODC* also led to significantly reduced concentrations of polyamines (putrescine, spermidine and spermine), tyramine and phenolamides (caffeoylputrescine and dicaffeoylspermidine) with concomitant increases in concentrations of amino acids ornithine, arginine, aspartate, glutamate and glutamine. Root transcript levels of *S*-adenosyl methionine decarboxylase, *S*-adenosyl methionine synthase and spermidine synthase (polyamine synthesis enzymes) were reduced compared with vector controls, whilst transcript levels of arginine decarboxylase (putrescine synthesis), putrescine methyltransferase (nicotine production) and multi-drug and toxic compound extrusion (alkaloid transport) proteins were elevated. In contrast, expression of two other key proteins required for alkaloid synthesis, quinolinic acid phosphoribosyltransferase (nicotinic acid production) and a PIP-family oxidoreductase (nicotinic acid condensation reactions), were diminished in roots of *odc*-RNAi plants relative to vector-only controls. Transcriptional and biochemical differences associated with polyamine and alkaloid metabolism were exacerbated in *odc*-RNAi plants in response to different forms of shoot damage. In general, apex removal had a greater effect than leaf wounding alone, with a combination of these injury treatments producing synergistic responses in some cases. Reduced expression of *ODC* appeared to have negative effects upon plant growth and vigour with some leaves of *odc*-RNAi lines being brittle and bleached compared with vector-only controls. Together, results of this study demonstrate that ornithine decarboxylase has important roles in facilitating both primary and secondary metabolism in *Nicotiana*.

## Highlight

RNAi-mediated reduction of *ornithine decarboxylase* gene activity in tobacco has negative effects on plant growth and leads to widespread alterations in primary and secondary metabolism, particularly in wounded plants.

## Introduction

Terrestrial plants have been subjected to herbivory since their emergence onto land *ca*. 450 million years ago and a wide array of physical and chemical defence systems have evolved to provide protection and facilitate their reproduction in native environments (Labandeira, 1998; Wellman and Gray, 2000). Alkaloids represent a diverse grouping of such chemical defences, with many thousands of chemical structures distributed widely across the plant kingdom (Aniszewshi, 2015). Biosynthesis of alkaloids generally involves the diversion of amino acid precursors from primary into secondary metabolism via the action of decarboxylases and is often enhanced by exposure of plants to biotic and/or abiotic stress conditions (Shoji and Hashimoto, 2013a).

**Fig. 1.**
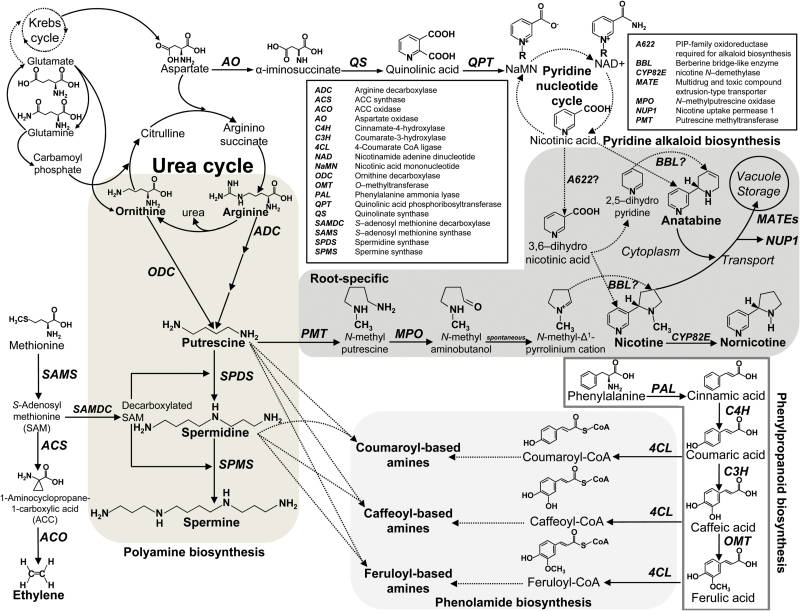
Schematic diagram of the biosynthesis of polyamines and connections with other metabolic pathways, including pyridine alkaloid, phenolamide and phenylpropanoid metabolism, in *N*. *tabacum* (adapted from Cane *et al.*, 2005; Onkokesung *et al.*, 2012; Shoji and Hashimoto, 2012; Winter *et al.*, 2015). Solid lines indicate defined steps, while dotted lines indicate undefined steps or steps including multiple reactions. Both pyridine alkaloid and polyamine/phenolamide metabolites share early biosynthetic steps, starting from arginine and ornithine to produce putrescine. The biosynthesis of alkaloids in tobacco occurs mainly in roots, while the phenolamide and phenylpropanoid pathways are present predominantly in above-ground (leaf) tissues. The final condensation reactions with nicotinic acid derivatives and *N*-methyl pyrrolinium cation are not yet clear; however, it is suggested that a PIP-family isoflavone reductase-like protein, A622, is involved in the late step of synthesis of the pyridine moiety prior to synthesis steps involving enzymes from a Berberine Bridge Like gene family. Several toxin transport proteins including MATEs and NUP1 are involved in vacuolar storage and transport of alkaloids to aerial tissues (Shoji *et al.*, 2009; Morita *et al.*, 2009; Hildreth *et al.*, 2011; Shitan et al., 2014a,b).

The genus *Nicotiana* (family Solanaceae) contains more than 75 species, native mainly to the Americas and mainland Australia, with representatives also on South Pacific islands and in southern Africa (Knapp *et al.*, 2004). The genus is well known for its production of a range of pyridine alkaloids, particularly nicotine, nornicotine, anabasine and anatabine, which are found at various concentrations in all *Nicotiana* species (Saitoh *et al.*, 1985). Acting as agonists on the nervous system of herbivores, both invertebrate and vertebrate, they discourage feeding and increase the rate of herbivore mortality and/or susceptibility to predatory attack (Steppuhn *et al.*, 2004). Figure 1 provides an overview of alkaloid biosynthesis in *N. tabacum* and its relationship with other aspects of putrescine metabolism.

Synthesis of the alkaloid nicotine has been reported to be energy demanding (Bush *et al.*, 1999) and diversion of nitrogen from primary metabolism, growth and reproduction into synthesis of this defence compound can also have fitness costs, as demonstrated in *N. attentata* growing in native environments (Baldwin *et al.*, 1990; Baldwin and Ohnmeiss, 1994; Ohnmeiss and Baldwin, 2000). Experiments with cultivated *N. tabacum*, and also native species *N. sylvestris* and *N. attenuata*, showed that damage to aerial tissues led to an increase in nicotine content of leaves within several days of wounding (Saunders and Bush, 1979; Baldwin, 1989; Baldwin and Ohnmeiss, 1993; Baldwin and Ohnmeiss, 1994; Sinclair *et al.*, 2004). Studies in *Nicotiana* have attributed the transmission of wound signals resulting from leaf damage and apex removal (topping) from aerial to root tissues to increased JA and reduced auxin levels, respectively (Baldwin *et al.*, 1994; Baldwin *et al.*, 1996; Shi *et al.*, 2006). Recent reports indicate that a convergence of both JA and auxin cross-signalling networks is likely to operate at the molecular level *in vivo* through shared components of these transduction pathways (Pauwels *et al.*, 2010; Hentrich *et al.*, 2013; He and Zhao, 2015). Transcription of key structural genes required for alkaloid biosynthesis is regulated via the action of several transcription factors, including MYC2 and APETALA2/ETHYLENE RESPONSE FACTOR (AP2/ERF) types, which are themselves regulated by these hormones (reviewed in Dewey and Xie, 2013; Shoji and Hashimoto, 2013a). Alkaloid transport from roots to the aerial tissues occurs via the xylem system, with loading/unloading into the xylem and storage in leaf vacuoles involving several MULTIDRUG AND TOXIC COMPOUND EXTRUSION (MATE)-type transporters, as well as NICOTINE UPTAKE PERMEASE (NUP1) proteins (Morita *et al.*, 2009; Shitan *et al.*, 2009; Shoji *et al.*, 2009; Hildreth *et al.*, 2011; Shitan *et al.*, 2014a; Shitan *et al.*, 2014b; Kato *et al.*, 2014).

The diamine putrescine is an important intermediate precursor in the synthesis of higher amines, spermidine and spermine, which play important roles in metabolic, physiological and developmental processes in all living organisms (Fariduddin *et al.*, 2013; Kusano and Suzuki, 2015). In most plant species, putrescine can be synthesized from either ornithine or arginine via the activity of the decarboxylating enzymes, ORNITHINE DECARBOXYLASE (ODC) or ARGININE DECARBOXYLASE (ADC), respectively (Shoji and Hashimoto, 2013a; Michael, 2015). Plant polyamines exist predominantly as conjugates with hydroxycinnamic acids in the Solanaceae family, collectively described as phenolamides (Fig. 1; Smith *et al.*, 1983; Martin-Tanguy, 1985; Kaur *et al.*, 2010; Onkokesung *et al.*, 2012). Such conjugated polyamines have been reported to occur throughout the plant kingdom and appear to have roles in chemical defence as well as aspects of plant development (Kaur *et al.*, 2010; Fellenberg *et al.*, 2012; Onkokesung *et al.*, 2012).

In many solanaceous genera, putrescine is also an important precursor for alkaloid synthesis, including nicotine and nornicotine (reviewed in Dewey and Xie, 2013; Shoji and Hashimoto, 2013a). Synthesis of nicotine involves the condensation of a nicotinic acid-derived pyridine ring, sourced from the pyridine nucleotide cycle, with a pyrrolidine ring derived from putrescine (Dewey and Xie, 2013; Shoji and Hashimoto, 2013a). A further step involving the *N*-demethylation of nicotine is the primary means of producing nornicotine (Siminszky *et al.*, 2005; Lewis *et al.*, 2008; Lewis *et al.*, 2010). Anatabine, the other main alkaloid in *N. tabacum*, is derived entirely from two molecules of nicotinic acid (Leete and Slattery, 1976; Leete, 1992). Using antisense and RNAi methodology, our previous studies indicated that marked reductions in *ODC*, but not *ADC*, transcript levels had a marked effect on the capacity of transgenic *N. tabacum* to synthesize nicotine (Chintapakorn and Hamill, 2007; DeBoer *et al.*, 2011a). In the current study, utilizing T_2_ generation plants of *N. tabacum* homozygous for an introduced *odc*-RNAi construct (DeBoer *et al.*, 2011a), we undertook a detailed analysis of the effects of down-regulating *ODC* upon the production of amines and associated pools of amino acids, as well as the changes in defence chemistry and components of the associated root transcriptome.

## Materials and methods

### Plant material

Homozygous T_2_ plants were generated from transgenic *N. tabacum* (SC 58 variety, AABB genotype; Chaplin, 1966; Cane *et al.*, 2005) lines *At-Nt odc*-RNAi-3 and *At-Nt-odc*-RNAi-4 plants, which were described fully in DeBoer *et al.* (2011a). Comparable T_2_ homozygous plants containing the T-DNA insert from an empty pART27 vector (vector-only control; VC) were used as a transformation control. These plants were identical in growth habit and morphology to those of non-transgenic parental line SC 58. Seeds of all lines were surface-sterilized and germinated *in vitro* on Murashige and Skoog (MS) agar plates containing 3% sucrose and 75 μg mL^−1^ kanamycin sulphate according to Chintapakorn and Hamill (2003) and maintained in a 25 °C/16h photoperiod and allowed to grow for ~4 weeks before transfer to 250mL glass jars containing 50mL of agar-solidified MS medium. Two weeks later seedlings (~4–6 leaf stage) were placed in rockwool blocks and transferred to communal hydroponic trays to acclimatize for a further 2 weeks before being placed in individual hydroponic containers each containing 200mL of full strength Hoagland’s medium, formulated as described previously (Cane *et al.*, 2005). Hydroponic chambers were randomly distributed on a communal bench at 20cm intervals in an insect-proof (PC2) glasshouse at 25±2 °C and plants grown under uniform supplemental fluorescent lighting and a 16/8h photoperiod, with bi-weekly media changes and daily liquid replenishment to 200mL with deionized water as described in previous work (DeBoer *et al.*, 2009; DeBoer *et al.*, 2011a; DeBoer *et al.*, 2013). For further seed production, additional seedlings of each line (~4–6 leaf stage) were grown on a common damp mat in the same greenhouse at 25±2 °C, under ambient lighting, in 250mL pots of compost (3 parts seed raising mix : 1 part perlite) containing a single application of controlled release complete fertilizer (Osmocote®, 15g L^−1^ of compost mix) as recommended by the manufacturer (Scotts Australia Pty Ltd).

### Plant treatments

After 4 weeks growth in hydroponics, transgenic *N. tabacum* plants (~10–12 leaf stage) either remained non-wounded (C) or were mechanically injured in one of three ways as follows. (1) Designated ‘W’ for wounded; a fabric pattern wheel was drawn across the lamina twice on each side of the mid-vein of the two uppermost (>50%) expanded leaves to simulate insect attack (Ohnmeiss and Baldwin, 1994). (2) Designated ‘A’ for apex removal; a sharp scalpel blade was used to remove the shoot apex and young leaves (less than 50% expanded) to simulate ‘topping’ (Saunders and Bush, 1979; Bush *et al.*, 1999). (3) Designated ‘W+A’; a combination of both damage treatments was used. Unless otherwise stated, chemical analysis was performed using wounded leaves or the two leaves located immediately below the apex removal point. Tissues were harvested 24h or 7 days after treatment for RNA or metabolite analysis, respectively, in line with previous work from this laboratory (Cane *et al.*, 2005; DeBoer *et al.*, 2009; DeBoer *et al.*, 2011a; DeBoer *et al.*, 2013). In non-damaged control plants, cotton thread was tied loosely around the petiole of phyllotactically equivalent leaves on day 0, with these leaves being harvested for analysis at the same time point as in damaged plants.

### Targeted analysis of leaf and root primary and secondary metabolites

Concentrations of amino acids, amines, alkaloids and phenolamides were determined using portions of homogeneous powdered tissue that had previously been freeze-dried for a minimum of 48h. One hundred milligrams of freeze-dried ground leaf or root powder was pre-weighed and aliquoted into 1.5mL Eppendorf tubes containing a sterile stainless steel ball to aid extraction for subsequent metabolite analyses. Alkaloids and phenolamides were extracted from leaf and root samples using an optimized 40% methanol extraction method described by Gaquerel *et al.* (2010). Amino acids were analysed and quantified by LC-MS/MS. Samples were prepared as reported above for alkaloid analysis and aliquots of the supernatant were diluted and analysed as described by Jander *et al.* (2004). Amines (putrescine, spermidine, spermine and tyramine) were extracted using an optimized hydrochloric and boric acid extraction and supernatant aliquots were analysed as *ortho*-phthaldialdehyde/ethanethiol/fluorenylmethoxycarbonyl derivatives as described by Fellenberg *et al.* (2012). Concentrations of amino acids, amines, alkaloids and phenolamides were quantified relative to known concentrations of standards and are graphically presented per milligram dry weight of tissue sample that was extracted.

### Quantitative real time PCR

Total RNA was isolated from leaf and root tissues snap-frozen in liquid N_2_ using a hot phenol method adapted from Verwoerd *et al.* (1989) and previously found to be suitable for extraction of high quality RNA from both leaf and root tissue of *Nicotiana* species (Cane *et al.*, 2005; DeBoer *et al.*, 2011a). DNase treated RNA was reverse transcribed with Superscript III reverse transcriptase (Invitrogen) using oligo (dT) 18 following the manufacturer’s recommendations. Quantitative RT-PCR (qRT-PCR) was performed with approximately 150ng of cDNA on a Lightcycler 480 real-time instrument (Roche) using SensiMix^TM^ SYBR no-ROX (Bioline) following the manufacturer’s recommendations. Previously published gene-specific primers (Shoji *et al.*, 2008; Shoji *et al.*, 2010; Schmidt and Delaney, 2010; Shoji and Hashimoto, 2011) were used, with slight modifications where stated, so that each primer pair combination produced an amplicon of ~100bp representing all known respective gene family members (see Supplementary Table S1 at *JXB* online). Results were obtained from analysis of three independent samples per treatment, each containing three technical replicates. Data were analysed using the 2^–ΔΔCT^ method (Livak and Schmittgen, 2001) and are presented as the fold change in gene expression for that particular gene family, each normalized to elongation factor 1α (EF1α) and relative to the corresponding non-wounded VC at time-zero.

### Statistical analysis

All statistical tests were performed using R 3.1.2 (http://www.r-project.org/) and R-Studio (v.0.98.976, http://www.rstudio.com/).

## Results

### Down-regulation of *ODC* reduces concentrations of amines in leaves and roots

**Fig. 2.**
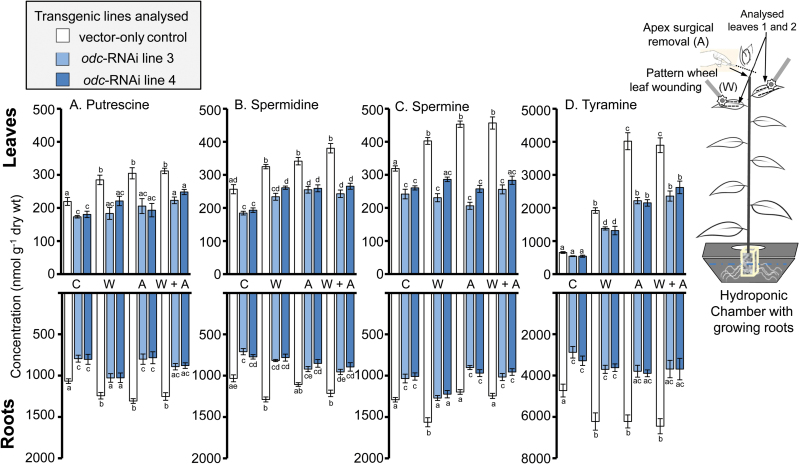
Down-regulation of *ODC* influences levels of amines in leaf and root tissues of non-wounded and wounded *N. tabacum* plants. Four weeks after transfer to hydroponics, VC and *odc*-RNAi transgenic plants were left non-wounded (C), mechanically damaged with a pattern wheel applied to the two uppermost expanded leaves (W), wounded by removing (‘topping’) the apical region containing leaves that were less than 50% expanded (A), or subjected to a combination of both wounding treatments (W+A). Phyllotactic equivalent leaves from untreated plants that were not damaged were analysed as a wounding control comparison. Mean (±SE) putrescine (A), spermidine (B), spermine (C), and tyramine (D) content (nmol) per gram dry weight (wt) in the two uppermost expanded leaves (top panel) or root tissues (bottom panel) harvested from *odc*-RNAi transgenic *vs*. VC plants 7 d post-wounding and quantified by UHPLC. Significantly different concentrations of amines among the genotypes and treatment groups at *P*<0.05 were determined by two-way analysis of variance (ANOVA) followed by Tukey’s honest significant difference (HSD) test and are indicated by different letters (*n*=4).

Concentrations of polyamines in both leaves and roots, and tyramine in roots, were significantly lower in non-wounded *odc*-RNAi transgenic plants compared with corresponding tissues of non-wounded VC plants (Fig. 2A–D). Concentrations of each amine in VC plants increased following wounding treatments, being particularly evident for tyramine, which showed significant increases of ~250–500% in leaf tissues of plants damaged by leaf wounding and apex removal, respectively (Fig. 2A–D). In general, *odc*-RNAi transgenics showed a reduced capacity to increase concentrations of these amines in leaves and roots in response to damage of aerial tissues, and no cases were observed where concentrations were elevated significantly above levels present in corresponding tissues of non-wounded VC plants (Fig 2A–D).

### Analysis of pyridine alkaloids and phenolamides in *odc*-RNAi plants

**Fig. 3.**
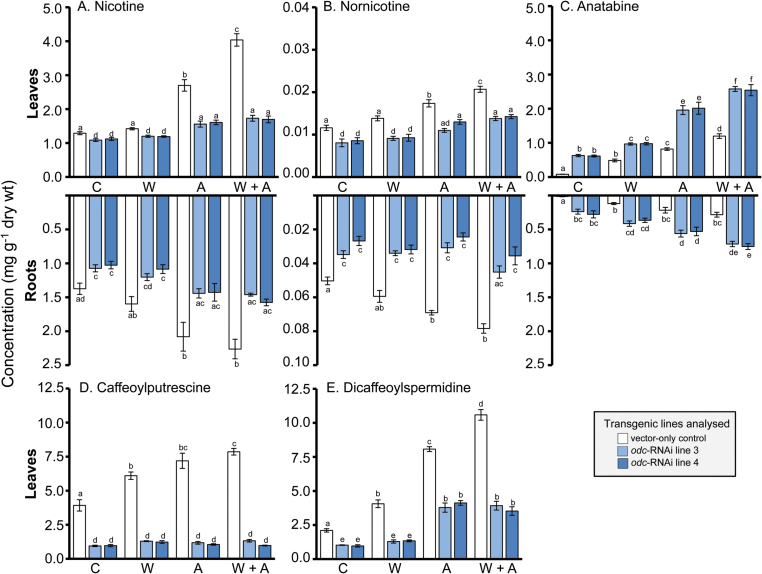
Down-regulation of *ODC* affects capacity to accumulate pyridine alkaloids and phenolamides in non-wounded and wounded *N. tabacum* plants. Leaves of VC and *odc*-RNAi transgenic plants were treated and harvested 7 d post-treatment as described in Fig. 2. Shown are the mean (±SE) concentrations of nicotine (A), nornicotine (B), and anatabine (C) in the two uppermost expanded leaves (top panel) and root tissue (bottom panel) from *odc*-RNAi *vs*. VC plants, and concentration of caffeoylputrescine (D) and dicaffeoylspermidine (E) in the two uppermost expanded leaves. Roots were analysed for phenolamides but these were present at trace levels and not quantifiable in most cases. Significant differences (*P*<0.05) between the lines and treatment groups were determined by two-way ANOVA followed by Tukey’s HSD test and are indicated by letters (*n*=4). A, apex removed; C, control; W, leaf wounded; W+A, wound plus apex removal.

As anticipated (Hamill *et al.*, 1986; Parr and Hamill, 1987), pyridine alkaloid analysis of upper leaves and roots of non-wounded *N. tabacum* VC plants revealed mainly nicotine, with lower concentrations of anatabine and nornicotine also being present (Fig. 3A–C). The two uppermost expanded leaves of non-wounded VC plants contained ~1.3mg nicotine g^−1^ dwt, with similar concentrations of nicotine observed in the roots (Fig. 3A). Nornicotine levels were 100-fold lower than nicotine in these leaves (~12 µg g^−1^ dwt) and 25-fold lower in roots (50 µg g^−1^ dwt; Fig. 3B). Anatabine levels were also low in non-wounded VC plants, being ~50 µg g^−1^ dwt in leaves and at trace levels in roots (Fig. 3C). Consistent with previous observations of T_1_ *odc*-RNAi transgenics (DeBoer *et al.*, 2011a), analysis of alkaloid concentrations in non-wounded T_2_ *odc*-RNAi plants revealed markedly different profiles from correspondingly non-wounded VC plants. Nicotine concentrations were significantly reduced in roots and uppermost expanded leaves of non-wounded *odc*-RNAi plants (Fig. 3A). Nornicotine concentrations in non-wounded *odc*-RNAi plants were also significantly reduced, dropping to ~8 µg g^−1^ dwt in leaves and ~35 µg g^−1^ dwt in roots (Fig. 3B). In marked contrast, anatabine concentrations were significantly elevated in leaves of non-wounded *odc*-RNAi plants, rising to ~600 µg g^−1^ dwt, which was >10-fold higher than in non-wounded VC plants. Unlike non-wounded VC plants, anatabine was also readily detectable in roots of non-wounded *odc*-RNAi plants (~200 µg g^−1^ dwt; Fig. 3C).

Wounding of VC plants had a stimulatory effect on the concentrations of all alkaloids in leaves and roots, with combined leaf wounding and apex removal causing the greatest increase in both leaves and roots of plants. In the latter treatment group, there was a ~3.5-fold increase in nicotine content of leaves (rising to ~4mg g^−1^ dwt) and a ~2-fold increase in nicotine content of roots (rising to ~2.4mg g^−1^ dwt); a ~2-fold increase in nornicotine concentrations (rising to ~20 μg^−1^ dwt in leaves and ~80 μg^−1^ dwt in roots); and a ~10-fold increase in anatabine concentrations (rising to ~1mg g^−1^ in leaves and ~250 μg^−1^ dwt in roots; Fig. 3A–C). Alkaloid analysis of *odc*-RNAi plants that had been subjected to either the apex removal or combined leaf-wounding and apex-removal treatment showed some capacity to increase concentrations of nicotine and nornicotine, but levels in both leaves and roots were never significantly higher than in comparable tissues of non-wounded VC plants (Fig 3A–B). On the other hand, anatabine concentrations were significantly elevated across all wounding treatments in *odc*-RNAi plants, representing an increase of 50–70% over that of similarly damaged VC plants. Thus, anatabine concentrations reached a maximum of ~2.5mg g^−1^ dwt in leaves and ~750 μg g^−1^ dwt in roots of *odc*-RNAi plants that experienced the combined apex removal and leaf wounding treatment, compared with ~1.2mg g^−1^ dwt in leaves and ~250 μg g^−1^ dwt in roots of comparable VC plants (Fig. 3C).

Wounding of VC plants produced significant increases in caffeoylputrescine and dicaffeoylspermidine concentrations compared with non-wounded counterparts (Fig. 3D–E). The stimulatory effects on phenolamide concentrations varied in magnitude in relation to the damage inflicted, with leaf-only wounded < apex removal < combined leaf wounding and apex removal treatment (Fig. 3D–E). Concentrations of caffeoylputrescine and dicaffeoylspermidine were reduced significantly in leaves of non-wounded *odc*-RNAi plants relative to non-wounded VC plants (Fig. 3D–E). Unlike VC plants, wounding produced no stimulatory effect upon caffeolyputrescine concentrations in the *odc*-RNAi lines (Fig. 3D). Dicaffeoylspermidine concentrations were elevated 3- to 4-fold in *odc*-RNAi plants that experienced apex removal, but total levels remained significantly lower compared with similarly damaged VC plants (Fig. 3E).

### Amino acid analysis of *odc*-RNAi *versus* vector control plants

**Fig. 4.**
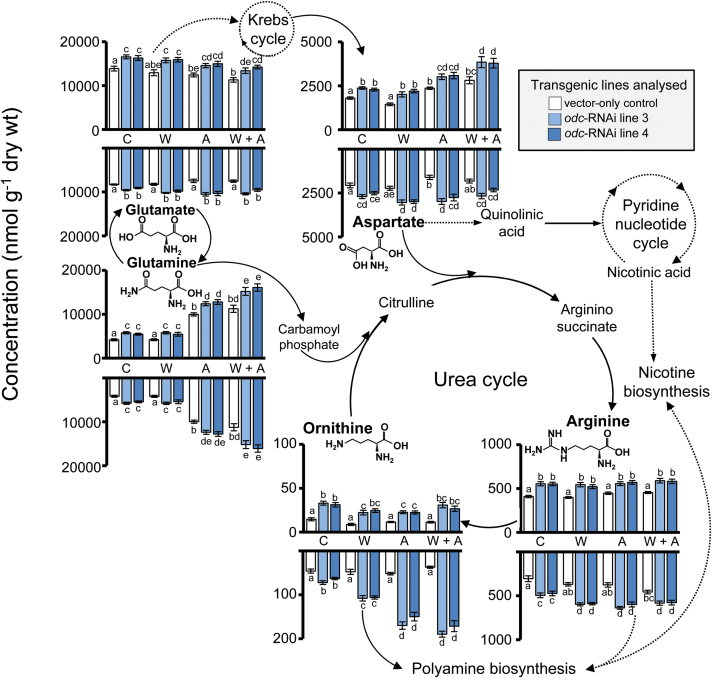
Down-regulation of *ODC* affects accumulation of inter-related amino acids in non-wounded and wounded *N. tabacum* plants. VC and *odc*-RNAi transgenic plants were treated and harvested 7 d post-treatment as described in Fig. 2. The schematic diagram depicts a simplified version of the metabolic connections between various related amino acids, pyridine nucleotide cycle components, polyamines (putrescine and spermidine), and pyridine alkaloid (nicotine) metabolic pathways. Solid lines indicate single defined steps, while dotted lines indicate steps composed of multiple reactions. Mean (±SE) arginine, aspartate, glutamate, glutamine, and ornithine content (nmol) per gram dry weight (wt) in the two uppermost expanded leaves and roots (top and bottom panels, respectively, in each pair of graphs) from vector-only *vs*. *odc*-RNAi transgenic plants. Significantly different levels of amino acids among the lines at *P*<0.05 were determined by two-way ANOVA followed by Tukey’s HSD test and are indicated by different letters (*n*=4).

Silencing of *ODC* resulted in a significant increase (2- to 3-fold) in baseline levels of ornithine in leaf and root tissues of non-wounded *odc*-RNAi transgenics compared with VC plants (Fig. 4). Ornithine concentrations in leaf tissues were not increased further in response to wounding in either VC or *odc*-RNAi plants. Roots of VC plants also did not show an increase in ornithine concentrations as a result of any of the wounding treatments. However, in the roots of *odc*-RNAi transgenics, ornithine concentrations were significantly enhanced (~3-fold) by leaf wounding alone and by apex removal (5- to 6-fold) relative to similarly treated VC plants (Fig. 4). Interestingly, *odc*-RNAi plants also showed significant increases in baseline concentrations of arginine in leaf (20–25%) and root (30–40%) tissues compared with VC plants. Wounding did not significantly alter arginine concentrations in leaves of VC plants but there was a significant, albeit <2-fold, increase in arginine concentration of roots in response to the combined leaf wounding and apex removal treatment (Fig 4). Wounding increased arginine concentrations further in root, but not leaf, tissues of *odc*-RNAi plants relative to comparable VC controls (Fig. 4).

Baseline leaf and root glutamate concentrations were increased by 20–25% in *odc*-RNAi transgenics compared with VC counterparts (Fig. 4). Similarly, silencing of *ODC* resulted in higher baseline concentrations of glutamine relative to non-wounded vector plants. In both *odc*-RNAi and VC plants, glutamine levels did not increase in response to leaf wounding alone, but interestingly did increase ~2-fold in leaf tissues as a result of apex removal. Glutamine levels were 20–30% higher in these apex removed *odc*-RNAi plants than in corresponding VC plants (Fig. 4). Aspartate levels were generally 20–35% higher in non-wounded and wounded *odc*-RNAi plants, relative to correspondingly treated VC plants. In all genotypes, there was a 20–30% increase in aspartate levels of leaf, but not root, tissues of plants wounded by apex removal or combined with leaf damage relative to comparable non-wounded plants (Fig. 4).

### Analysis of key polyamine and alkaloid biosynthetic gene activity in roots of *odc*-RNAi *versus* vector control plants

**Fig. 5.**
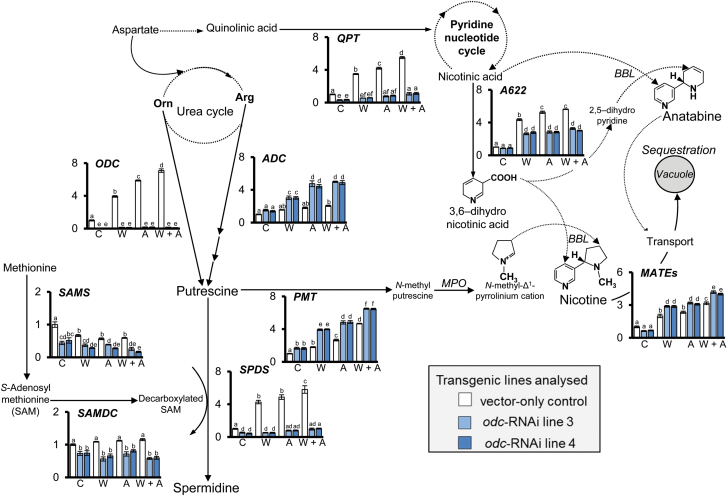
The effect of wounding on root transcript levels of key genes involved in polyamine and pyridine alkaloid synthesis in vector-only control and *odc*-RNAi plants. RNA was extracted from roots harvested 24h after wounding treatments as described in Fig. 2. Data shown in the schematic diagram represent the average (±SE) transcript abundance of designated alkaloid- and polyamine-associated genes normalized to the endogenous reference gene (EF1α) and relative to the non-wounded VC control at time-zero, which was arbitrarily designated as ‘1’. Significant differences (*P*<0.05) in levels of transcripts among VC and *odc*-RNAi plants were determined by two-way ANOVA followed by Tukey’s HSD test and are indicated by letters (*n*=4). For description of abbreviations see Fig. 1.

As alkaloid synthesis occurs predominantly in roots of *N. tabacum* (Dewey and Xie, 2013 and references therein), we undertook a detailed comparative analysis of transcript abundance relating to genes of alkaloid and polyamine metabolism in roots of *odc*-RNAi transgenics *vs.* VC plants. Consistent with previous studies involving wounded *N. tabacum* (Cane *et al.*, 2005; Shoji and Hashimoto, 2011 and references therein), analysis of VC plants 1 day post-treatment showed that leaf wounding only, apex removal only, and both leaf wounding and apex removal in combination generally caused progressively larger increases in transcript levels of genes involved in putrescine and spermidine synthesis (*ODC*, *ADC* and *SPDS*) and also alkaloid production and mobilization (*A622*, *PMT*, *QPT* and *MATE*) (Fig. 5). In contrast, transcript levels of other genes involved in polyamine synthesis either remained relatively constant (*SAMDC*) or were reduced (SAMS) in roots of wounded *vs.* non-wounded VC control plants (Fig 5).

Consistent with a previous study involving T_1_ *odc*-RNAi plants (DeBoer *et al.*, 2011a), *ODC* transcript levels were reduced by >95% in T_2_ *odc*-RNAi plants compared with VC plants, and did not increase significantly even after the combined wounding treatments (Fig. 5). The *ADC* wound response was enhanced in *odc*-RNAi plants where we observed significantly higher (~2- to 3-fold) levels of *ADC* transcript in roots of wounded *odc*-RNAi plants compared with VC counterparts, increasing in magnitude with plants damaged by leaf wounding < apex removal < combined leaf wounding and apex removal treatments (Fig. 5). Silenced *ODC* plants also displayed ~1.5- to 2-fold higher basal and wound-elicited levels of *PMT* transcript compared with VC plants. Basal transcript levels of *MATE* were similar in non-wounded VC and *odc*-RNAi plants. However, following wounding, we observed significantly higher levels of *MATE* transcripts in roots of *odc*-RNAi plants than in corresponding VC plants (Fig. 5). Interestingly, and unexpectedly, basal *QPT* transcript levels in roots of non-wounded *odc*-RNAi plants were found to be significantly lower (~3-fold) than in comparable VC plants. We observed even greater relative differences in roots of wounded *odc*-RNAi plants, where *QPT* transcript levels were 5- to 6-fold lower than in similarly wounded VC plants. Even in plants that experienced the most severe combined leaf wounding and apex removal treatment, *QPT* transcript levels of *odc*-RNAi plants were not significantly elevated above levels observed in non-wounded VC plants (Fig 5). Transcript levels of *A622* in roots of wounded *odc*-RNAi plants were also significantly lower than in VC counterparts, albeit the magnitude of reduction was much less pronounced than was observed for *QPT* (Fig. 5).

Transcript levels of genes encoding polyamine-related enzymes were also clearly altered in *odc*-RNAi plants compared with VC plants. *SPDS* was significantly up-regulated in roots in response to leaf wounding (~5-fold) and/or apex removal (6- to 7-fold) in VC plants, compared with non-wounded counterparts. A marked reduction (~50%) in relative baseline *SPDS* transcript levels was observed in non-wounded *odc*-RNAi plants compared with VC plants. This difference was further exacerbated in wounded *odc*-RNAi plants, with *SPDS* transcript levels being 75–80% lower than that of similarly wounded VC plants. Silencing of *ODC* also resulted in ~1.5- to 2-fold reductions in relative *SAMS* and *SAMDC* transcript levels in both non-wounded and wounded *odc*-RNAi-plants, compared with appropriate VC plants (Fig. 5).

### Effects of silencing *ODC* upon the spatial distribution of polyamine, alkaloid and phenolamide metabolites in decapitated *N. tabacum* plants

**Fig. 6.**
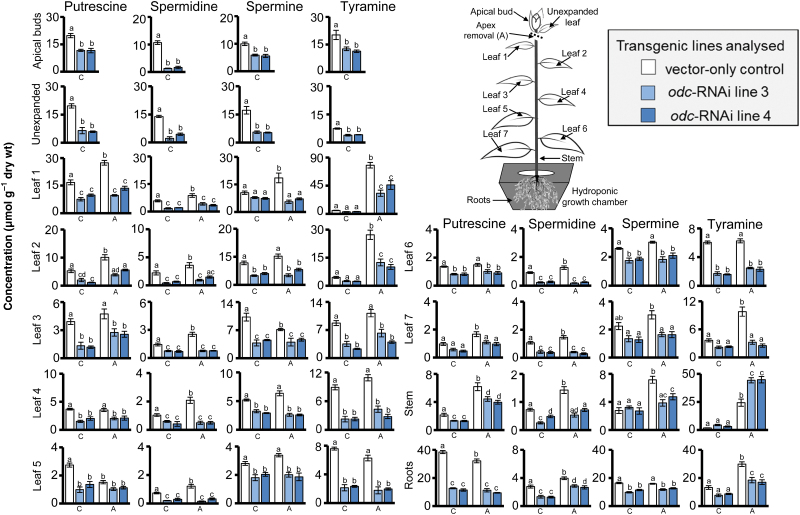
The spatial distribution of amines in non-wounded and wounded vector-only control and *odc*-RNAi plants. Four weeks after transfer to hydroponics, pre-flowering VC and *odc*-RNAi transgenic plants were either left unwounded (C) or were wounded by removing the apex, along with leaves less than 50% expanded (A). Different tissues were harvested and quantified for amines 7 d post-treatment. Mean (±SE) putrescine, spermidine, spermine, and tyramine content (µmol) per gram dry weight (wt) in apical buds, leaf (unexpanded through to leaf 7), stem and root tissue from *odc*-RNAi transgenic plants *vs*. VC plants. Significant differences (*P*<0.05) among plant lines were determined by two-way ANOVA followed by Tukey’s HSD test and are indicated by letters (*n*=4). Unexpanded leaves in non-wounded plants were less than 50% expanded at the time of harvest.

**Fig. 7.**
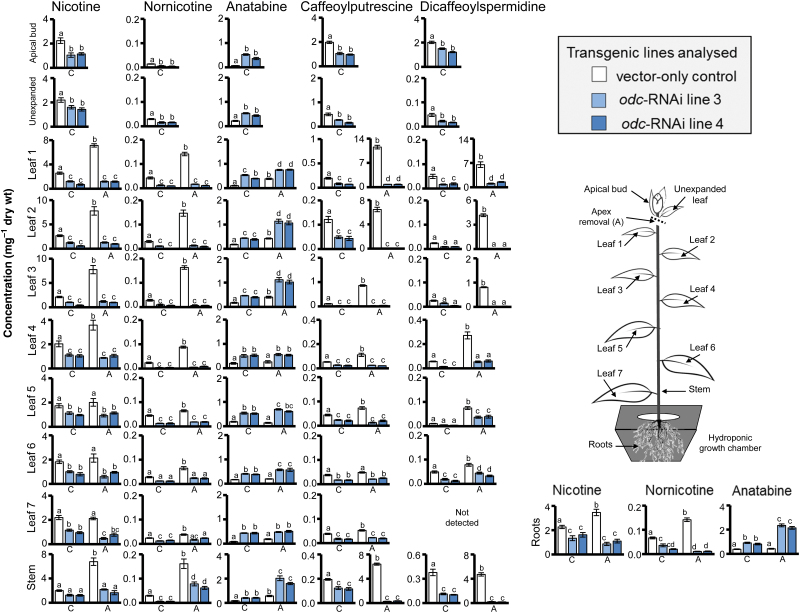
Levels of pyridine alkaloids and phenolamides in tissues of non-wounded and wounded vector-only control and *odc*-RNAi plants. Plants were either left unwounded (C) or had their apex removed (A) and tissues were harvested 7 d post-treatment as described in Fig. 6. Data represent mean (±SE) concentrations (mg) per gram dry weight (wt) of nicotine, anatabine, and nornicotine (pyridine alkaloids) and caffeoylputrescine and dicaffeoylspermidine (phenolamides) in apical buds, leaf (unexpanded through to leaf 7), stem, and root tissue from VC *vs*. *odc*-RNAi transgenic plants. Letters represent significantly different (*P*<0.05) concentrations of metabolites determined by two-way ANOVA and followed by Tukey’s HSD test among *odc*-RNAi and VC plants (*n*=4). Unexpanded leaves in non-wounded plants were less than 50% expanded at the time of harvest.

Experiments were performed to assess the effects of removing plant apices upon spatial distribution of polyamines, pyridine alkaloids and phenolamides throughout the plant (Figs 6–7). One week after apex removal, older leaves located progressively lower on the stem of plants, together with the stem tissue and roots of each plant, were analysed and compared with levels of these metabolites in phyllotactic equivalent tissues of non-wounded plants. In concurrence with previous experiments noted above, apical bud tissues, leaves, stem and root tissues from non-wounded *odc*-RNAi transgenic plants contained significantly lower concentrations of polyamines, tyramine, nicotine/nornicotine and phenolamides, compared with equivalent tissues in VC plants (Figs 6–7). The capacity to increase polyamines, tyramine, nicotine/nornicotine and phenolamide concentrations in response to apex removal was also significantly compromised in *odc*-RNAi plants compared with VC plants with the largest differences between both groups of plants being detected in upper (younger) leaves, as well as in the stem and roots. Tyramine concentrations were also lower in leaves and roots of wounded *odc*-RNAi lines than in VC plants but, interestingly, the converse was true in stem tissues where levels were ~2-fold higher in wounded *odc*-RNAi lines compared with similarly wounded VC plants (Fig. 6). Consistent with our previous study involving T_1_ *odc*-RNAi transgenic plants (DeBoer *et al.*, 2011a), the present study found that anatabine concentrations were significantly elevated in roots and leaves of T_2_ *odc*-RNAi plants, both non-wounded and wounded, compared with corresponding tissues of VC plants (Fig. 7).

### Effects of silencing *ODC* upon growth and flowering in *N. tabacum*

**Fig. 8.**
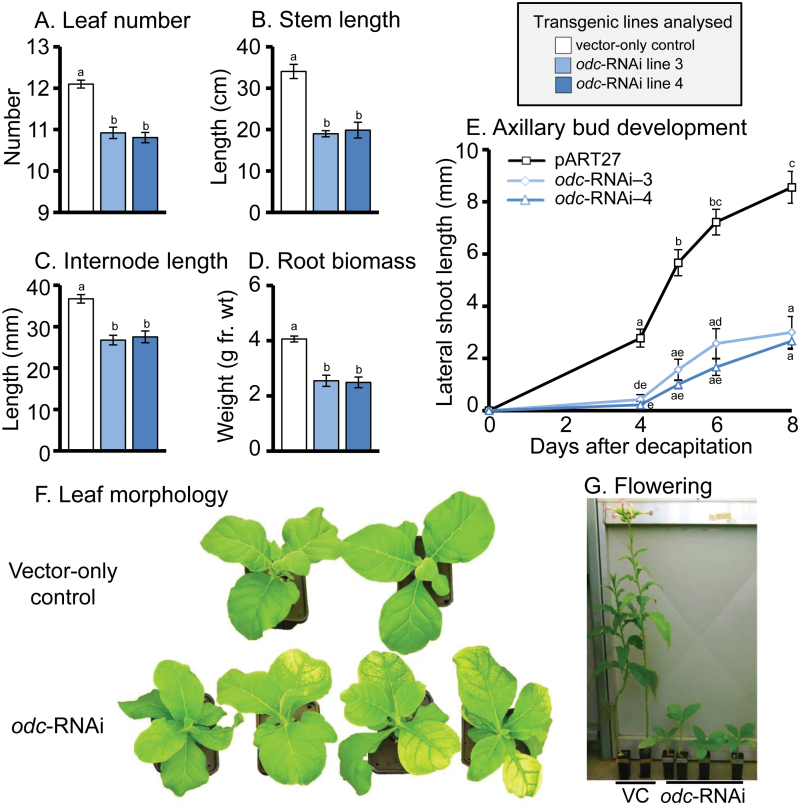
Growth and morphological traits in vector-only control and *odc*-RNAi plants. Seedlings cultured on MS medium *in vitro* for 6 weeks (4–6 leaf stage) were transferred to either hydroponics (A–F) with continual nutrient replenishment or transplanted into compost (G) containing an initial application of Osmocote® controlled release complete fertiliser. Five weeks after plants were transferred to hydroponics, leaf number (A), stem length (B), internode length (C), root biomass blotted to remove extra liquid (D) and leaf morphology (F) were recorded from non-wounded VC and *odc*-RNAi transgenic plants. (Note the presence of chlorotic sections on leaves of *odc*-RNAi plants, unlike VC plants as shown in panel E). Plants were then decapitated to a uniform height of 10cm, all remaining leaves were removed, and the average length of the top three axillary buds emerging following decapitation was determined over the following 8 days (E). ​Significant differences (*P*<0.05) between lines, represented by letters, were calculated by one-way or two-way ANOVA followed by Tukey’s HSD test (*n*=4). VC plants that were transferred to compost at the ~4–6 leaf stage, with the aim of collecting seeds after self-fertilization, were observed to be approximately 60–80cm high after a further 12 weeks cultivation, with evidence of inflorescence formation or flowers beginning to open. In contrast, *odc*-RNAi plants were much shorter in height (10–25cm) with no signs of inflorescence development at this time point (G; lower senescent leaves removed prior to photography). The *odc*-RNAi plants shown here did flower, however, after a further 8–12 weeks of greenhouse cultivation, with heights of plants at flowering being approximately half that of the VC controls when they commenced flowering. Overall floral morphology and self-fertility were not noticeably altered in these *odc*-RNAi transgenics compared with vector-only control plants (data not shown). fr. wt: fresh weight.

In a separate study, Nölke *et al.* (2005) reported that the use of immuno-modulation to inhibit ODC enzymatic activity in transgenic *N. tabacum* led to a decrease in levels of all three polyamines. Morphological changes were also observed, including stunted plants with elongated leaves that produced smaller and fewer flowers. In our previous experiments involving T_1_ offspring of transgenic plants, we did not observe an obvious negative effect upon phenotype in hydroponically grown plants containing the *odc*-RNAi construct (DeBoer *et al.*, 2011a). However, in the present study, using T_2_ offspring homozygous for the introduced empty pART27 vector and *odc*-RNAi constructs, careful observation did reveal a number of negative effects upon leaf morphology, growth and reproductive parameters in hydroponically grown *odc*-RNAi transgenics compared with VC plants (Fig. 8). These alterations became progressively more obvious with age and, although much less pronounced, were reminiscent of the effects observed by Nölke *et al.* (2005). Thus, at ~11 weeks old, hydroponically grown *odc*-RNAi plants had produced on average, one fewer leaf than their equivalently aged, similarly cultivated VC counterparts (Fig. 8A). This was accompanied by reductions in stem length (Fig. 8B), internode length (Fig. 8C), root biomass (Fig. 8D), and rate of axillary bud emergence and outgrowth following decapitation of plants to 10cm in height (Fig. 8E). We also noticed that leaves of hydroponically grown *odc*-RNAi plants displayed tendencies for sporadic bleaching and occasional chlorosis (Fig. 8F) of entire leaves, which were slightly epinastic and brittle compared with leaves of hydroponically grown VC plants (Fig 8F). Although these alterations in leaf morphology bore some resemblance to classic symptoms of mineral deficiencies, separate growth experiments showed they were not prevented by more frequent replenishment of the Hoagland’s nutrient medium in each container (three times per week) or by separately altering the concentrations of nitrate, iron or manganese over a range from one-quarter strength to double strength that of normal Hoaglands, with twice weekly changes in medium (data not shown). In contrast to hydroponically grown plants, seedlings transplanted to compost, with the aim of generating sufficient seed for further studies, showed *odc*-RNAi lines to be markedly slower growing and delayed in their time of flowering compared with VC plants (Fig. 8G). We hypothesize that these differences in growth capacity between hydroponically and soil grown sibling *odc*-RNAi transgenic plants reflects a reduced capacity to recover essential nutrients from the compost mix, likely due to diminished root system functionality compared with VC counterparts. Further analysis is ongoing to examine in detail this aspect of *odc*-RNAi transgenics *vs*. VC and wild-type plants of *N. tabacum*.

## Discussion

Although *N. tabacum* has been used for many years as a model system to study wound-associated alterations in alkaloid biosynthesis and transport from roots to aerial tissues, (for recent reviews see Dewey and Xie, 2013; Wang and Bennetzen 2015), the relationship between ‘primary’ and ‘secondary’ metabolism *in vivo* remains unclear, particularly in wounded plants. In the present study, we examined the broader metabolic consequences of down-regulating *ODC* transcript levels in *N. tabacum*, comparing non-wounded and wounded lines homozygous for an *odc*-RNAi construct with a control line homozygous for VC T-DNA, all derived from the transgenic plants described in the study of DeBoer *et al.* (2011a). Higher ornithine concentrations in *odc*-RNAi transgenics, relative to that of VC plants, was not unexpected given their reduced capacity to produce *ODC* transcript which, as shown previously, results in diminished ODC activity (DeBoer *et al.*, 2011a). As illustrated in Fig. 1, and discussed recently (Majumdar *et al.*, 2013; Winter *et al.*, 2015; Majumdar *et al.*, 2016), there is a close relationship between ornithine metabolism, catabolism of glutamate and the synthesis of arginine in plants. It is possible therefore that such elevated concentrations of ornithine in *odc*-RNAi transgenics may have led directly to increased rates of arginine synthesis as well as a build-up of glutamate in tissues that, in turn, led to higher concentrations of glutamine. It is possible too that increased concentrations of aspartate in *odc*-RNAi transgenics, relative to VC plants, is directly linked to the diminished levels of *QPT* transcript in these plants, discussed in more detail below. Further work is thus warranted to examine both gene transcript levels and enzyme activities of the proteins responsible for conversion of glutamate–glutamine and glutamate–ornithine–arginine (Page *et al.*, 2012) and also aspartate–quinolinic acid and additional key enzymes associated with NAD synthesis (Katoh *et al.*, 2006; Noctor *et al.*, 2006).

Co-ordinated up-regulation of many genes encoding enzymes involved in alkaloid production, some also with important roles in primary metabolism such as *ODC* and *QPT* (Sinclair *et al.*, 2000; DeBoer *et al.*, 2011a; Ryan *et al.*, 2012), has been reported in several *Nicotiana* species (e.g. Goossens *et al.*, 2003: Sinclair *et al.*, 2004; Cane *et al.*, 2005; Shoji *et al.*, 2008) and involves concerted action of multiple JAZ-, MYC2-, and AP2/ERF-type regulatory proteins (Shoji *et al.*, 2008; Shoji *et al.*, 2010; DeBoer *et al.*, 2011b; Shoji and Hashimoto, 2011; Zhang *et al.*, 2012; Shoji and Hashimoto, 2012; Sears *et al.*, 2014; Shoji and Hashimoto, 2013b; Shoji and Hashimoto, 2015; Yang *et al.*, 2015). In previous experiments in which *PMT* and *ADC* genes were strongly down-regulated in *N. tabacum* hairy roots using an antisense approach, transcript levels of non-targeted alkaloid biosynthesis genes were generally similar in antisense lines compared with vector controls (Chintapakorn and Hamill, 2003; Chintapakorn and Hamill, 2007). Thus, our observations in the current work that *PMT* and MATE transcripts were significantly higher in roots of wounded *odc*-RNAi plants compared with similarly treated VC plants, whilst *A622* and *QPT* transcripts were significantly lower, were not anticipated. These observations may indicate the existence of one or more hitherto undefined additional regulatory control mechanism(s) controlling the synthesis of defensive alkaloids in *N. tabacum*, particularly in response to wounding. One or more mechanism(s) linking ornithine metabolism directly with pyridine alkaloid biosynthetic capacity in *Nicotiana* may also help explain previous observations whereby over-expression of a yeast *ODC* gene led to both elevated putrescine levels and increased concentrations of nicotine in hairy roots of *N. rustica* (Hamill *et al.*, 1990). Conversely, reduced transcript levels of *QPT*, *A622* and possibly other genes encoding key enzymes required for production and incorporation of the pyridine ring of anabasine may have contributed to the reduced ability of *odc*-RNAi transgenic plants of *N. glauca* to produce this alkaloid, compared with vector controls, in response to the removal of plant apices (DeBoer *et al.*, 2013). The biochemical nature of such regulatory mechanism(s) that may link primary and alkaloid specialized metabolism in *Nicotiana* remains speculative at the current time but it may be pertinent to note that in addition to being an important intermediate for various catabolic reactions, ornithine has been proposed to be an important signalling molecule that plays a key role in controlling synthesis of associated amino acids and polyamines (Majumdar *et al.*, 2013; Majumdar *et al.*, 2016). Also of relevance here may be the recent discovery of an miRNA decoy that accumulates in roots of *N. tabacum* in response to apex removal, leading to sequestration of an miRNA that targets *QPT*, thereby enabling increased levels of *QPT* transcript levels in roots of wounded plants (Li *et al.*, 2015). The use of RNAi and other approaches to alter gene expression will be valuable in exploring further the links between ornithine and alkaloid metabolism in a range of *Nicotiana* species and possibly also other plants that produce specialized metabolites derived from nicotinic acid (reviewed in Ashihara *et al.*, 2015)

Deployment of chemical defences is often allocated with a higher preference for reproductively significant tissues or younger tissues that are potentially more vulnerable to attack by herbivores (Baldwin *et al.*, 1990). Accordingly, in the current study the largest differences between alkaloid levels of wounded VC- and *odc*-RNAi transgenic plants were observed in leaves positioned in the upper half of plants. Markedly reduced concentrations of phenolamides were also found in the upper leaves of *odc*-RNAi plants compared with phyllotactic equivalent tissues of VC plants. These putrescine derivatives have recently been shown to be important defence agents against insect herbivores (Gaquerel *et al.*, 2010; Kaur *et al.*, 2010; Onkokesung *et al.*, 2012). Tyramine is also stimulated by wounding in *Nicotiana* (Guillet and De Luca, 2005; Kim *et al.*, 2011). Though not directly associated with putrescine or polyamine metabolism, it is noteworthy that *odc*-RNAi transgenics were also significantly less capable of increasing tyramine levels of leaves and roots following apex removal. At the present time we can only speculate as to the capacity of *odc*-RNAi transgenic *N. tabacum* plants to resist herbivory in an external environment. However, it is likely that they would be much more susceptible to insect attack than normal, as was reported previously for transgenic plants of *N. attenuata* in which overall levels of pyridine alkaloids were reduced as a result of down-regulation of the *PMT* gene (Steppuhn *et al.*, 2004).

With regards to effects of *ODC* gene manipulation upon polyamine metabolism, previous experiments have demonstrated a several-fold increase in putrescine content, but little change in overall levels of spermidine and spermine, in transgenic *Nicotiana* tissues engineered to over-express *ODC* from a variety of organisms including yeast (Hamill *et al.*, 1990), mouse (DeScenzo and Minocha, 1993) and the closely related solanaceous species *Datura stramonium* (Mayer and Michael, 2003). Experiments with cultured poplar cells also engineered to over-express mouse *ODC* reported similar results, with analysis of plant *ODC*, *ADC*, *SPDS*, and *SAMDC* transcript levels indicating that only the latter was significantly altered (reduced) in cells over-expressing the mouse *ODC* gene compared with vector controls (Page *et al.*, 2007). In the current study employing a contrasting approach in which native *ODC* was down-regulated in tobacco using an RNAi approach, diminished transcript levels of the polyamine synthesis genes *SAMDC*, *SAMS*, and *SPDS* in roots of *odc*-RNAi plants was observed compared with vector-only controls. Such reductions in transcript abundance in *odc*-RNAi transgenics may be directly linked with reduced putrescine supply and likely underpin the lower spermidine and spermine levels that we observed in these plants compared with their VC counterparts. On the other hand, an increase in the arginine concentration of these plants, together with elevated *ADC* transcript, is in line with previous observations suggesting compensatory increases in ADC activity in plants with lowered ODC activity (Nölke *et al.*, 2005; DeBoer *et al.*, 2011a). This may have facilitated maintenance of adequate baseline concentrations of putrescine and higher polyamines for essential primary and also specialized metabolism requirements and is consistent with suggestions that putrescine supply in plants is influenced biochemically by the metabolic flux of associated metabolites (Page *et al.*, 2012; Majumdar *et al.*, 2016).

In addition to changes in the metabolic profile of *ODC*-silenced plants, and unlike our earlier observations with plants of the T_1_ generation (DeBoer *et al.*, 2011a), we observed some negative effects upon plant growth and morphology in hydroponically grown T_2_ lines that were homozygous for the introduced *odc*-RNAi construct, compared with VC controls. Anomalies such as sporadic periodic production of chlorotic, bleached and brittle leaves may be linked to changes in photosynthetic machinery or nutritional deficiencies associated with reduced putrescine supply (Sfichi *et al.*, 2004; Ioannidis *et al.*, 2012). Other differences in these hydroponically grown *odc*-RNAi transgenics compared with vector-only controls, such as shorter internodes, reduced root biomass, slower rates of release of dormant axillary buds following decapitation, and also markedly reduced vigour and delayed onset of flowering in *odc*-RNAi plants when grown in soil, are broadly in line with morphological alterations that have been reported previously in polyamine mutants of tobacco (Malmberg and McIndoo, 1983); in *Nicotiana* plants treated with the ODC biochemical inhibitor difluoromethylornithine (DFMO; Burtin *et al.*, 1991); and in transgenics with immuno-modulated ODC (Nölke *et al.*, 2005). Such abnormalities may be indicative of spermine depletion rather than attenuation of putrescine or spermidine levels *per se* (Hanzawa *et al.*, 2000; Imai *et al.*, 2004; Nölke *et al.*, 2005). It will be of interest to determine whether introduction of a more distantly related *ODC* gene less likely to be down-regulated by the *Nicotiana odc*-RNAi construct used here, for example the *ODC* gene from yeast that was previously expressed in *N. rustica* (Hamill *et al.*, 1990), will lead to restoration of normal polyamine metabolism and patterns of growth in *odc*-RNAi transgenic lines of *N. tabacum*.

## Supplementary data

Supplementary data are available at *JXB* online.

Table S1. Sequences of gene-specific primers used for qRT-PCR

Supplementary Data

## Abbreviations:

A622: PIP-family oxidoreductase
ADC: arginine decarboxylase
JA: jasmonate
MATE: multi-drug and toxic compound extrusion
ODC: ornithine decarboxylase
PMT: putrescine methyltransferase
QPT: quinolinic acid phosphoribosyltransferase
RNAi: RNA interference
SAMDC: *S*-adenosyl methionine decarboxylase
SAMS: *S*-adenosyl methionine synthase
SPDS: spermidine synthase
VC: vector-only control.
