# Supplementary material for: Effects of down-regulating ornithine decarboxylase upon putrescine-associated metabolism and growth in Nicotiana tabacum L
Source: J Exp Bot. 2016 Apr 28;67(11):3367–81. doi: 10.1093/jxb/erw166 (PMC4892731; doi:10.1093/jxb/erw166)
Supplement: Supplementary Data [file supp_67_11_3367__index.html]

Effects of down-regulating ornithine decarboxylase upon putrescine-associated metabolism and growth in Nicotiana tabacum L — Effects of down-regulating ornithine decarboxylase upon putrescine-associated metabolism and growth in Nicotiana tabacum L. — Supplementary Data 

# Effects of down-regulating ornithine decarboxylase upon putrescine-associated metabolism and growth in *Nicotiana tabacum* L.

## Supplementary Data

Data files

- Supplemental\_Table\_S1.pdf - Supplementary Data
