# Supplementary material for: Effects of down-regulating ornithine decarboxylase upon putrescine-associated metabolism and growth in Nicotiana tabacum L
Source: J Exp Bot. 2016 Apr 28;67(11):3367–81. doi: 10.1093/jxb/erw166 (PMC4892731; doi:10.1093/jxb/erw166)
Supplement: Supplementary Data [file supp_erw166_Supplemental_Table_S1.pdf]

Supplementary Table S1.

Title: Effects of down-regulating ornithine decarboxylase (*ODC*) upon putrescine-associated metabolism and growth in *Nicotiana tabacum* L.

Authors: Heidi L. Dalton, Cecilia K. Blomstedt, Alan D. Neale, Ros Gleadow, Kathleen D. DeBoer, John D. Hamill

Sequences of gene-specific primers used for qRT-PCR. Previously published primers were utilised or modified in order to amplify a template of approximately 100 bp as close to the 3' end of the respective gene as possible.

Gene	Forward primer (5'–3')	Reverse primer (5'–3')	Reference
**EF-1a**	AAGCCCATGGTTGTTGAGAC	CGTTCTTGATAACACCAACAGC	Shoji et al. (2010)
**α – tubulin**	GCTACCATCAAGACTAAGCG	CTCCAGGAACAACAGTTGG	Shoji et al. (2008)
**UBC 2**	TGCAAAATCAGTGGAGTCC	TCTGAATTAGCTGGCGAG	Adapted from Schmidt and Delaney (2010)
**A622**	GGAAGACCCTCGAGAAGTTA	GAATGGCATATGGCCAAAT	Adapted from Shoji et al. (2010)
**ADC**	GCGGAGGAATTCTTGGAACA	CCACAAGGTAAGGCATGTTATG	Adapted from Shoji et al. (2010)
**MATE1/2**	TCTAAACAAGGAATGAAGGTGG	GACTTCTTTCCCCTTGCATA	Adapted from Shoji et al. (2010)
**ODC**	GTTAGCTGTTCTGTCGAATCG	AACAGTATCAAGAGCATCACA	Adapted from Shoji et al. (2010)
**PMT**	GCAGCATTCATTTTGCCATC	GACTCGATCTTGGTCCAATG	Adapted from Shoji et al. (2010)
**QPT1/2**	CTTCGTTGACTAGGATAATGC	CCCATTGATCAATTCTACAG	Shoji and Hashimoto (2011a)
**SAMDC**	CAGTGTCCGTGTCTGTCTCTG	ACAAATCCGAACGACACAGC	Shoji et al. (2010)
**SAMS**	CAAGGTGGACAGGAGTGGTG	TGCATAAGAAACCTGGACAATG	Shoji et al. (2010)
**SPDS**	CAGATGTAGCTGTAGGATACG	CAGCATCGTAAGTTCCTGC	Shoji et al. (2010)
